# Conceptual rethinking of whole-body perturbation-evoked potentials as a biomarker of extralemniscal sensory transmission, alertness and arousal

**DOI:** 10.3389/fnhum.2026.1825052

**Published:** 2026-06-05

**Authors:** Tim Lehmann, Makoto Miyakoshi, Jochen Baumeister

**Affiliations:** 1 Exercise Science and Neuroscience Unit, Department of Exercise and Health, Faculty of Science, Paderborn University, Paderborn, Germany; 2 Division of Child and Adolescent Psychiatry, Cincinnati Children's Hospital Medical Center, Cincinnati, OH, United States; 3 Department of Psychiatry and Behavioral Neuroscience, University of Cincinnati College of Medicine, Cincinnati, OH, United States

**Keywords:** extralemniscal system, perturbation-evoked responses, postural control, sensory processing, vertex potential

Unexpected perturbations to the postural state require the human central nervous system to rapidly detect and evaluate sudden sensory events. In this context, the perturbation-evoked potential (PEP) has commonly been interpreted as a cortical marker of postural error processing or a related posture-specific control mechanism (Varghese et al., 2017). However, several characteristic features of the PEP show phenomenologically notable resemblance to the well-established vertex potential (VP), a supramodal cortical response associated with salient and surprising sensory input across modalities (Somervail et al., 2025). This overlap motivates a broader perspective in which cortical responses to postural disturbance may potentially not reflect posture-specific processing, but rather a more general cortical mechanism related to the rapid detection of sensory change and the modulation of functional brain state.

In highly dynamic daily or sporting situations, effective compensation for unexpected disturbances of upright stance requires a rapid recruitment of an extensive sensorimotor network that coordinates context-specific motor outputs to preserve body alignment and joint stability (Johansson, 1991; Shumway-Cook and Woollacott, 2012; Varghese et al., 2017). The successful execution of these compensatory responses thereby depends on the functional integrity of multisensory integration processes across peripheral and central pathways, enabling the nervous system to rapidly encode and evaluate afferent information from somatosensory, vestibular and visual modalities. This integrated sensory representation provides the basis for generating adaptive motor commands that ensure the stabilization or reallocation of the body's center of mass within the boundaries of the base of support, preventing misalignment of individual joints and risk of falling (Johansson, 1991; Horak et al., 1997; Shumway-Cook and Woollacott, 2012).

A comprehensive understanding of these complex control processes requires methodological approaches capable of capturing the rapid cortical responses that accompany external disturbances of posture. Among the neurophysiological methods available to investigate neural signatures of unexpected postural disturbances, commonly referred to as perturbation-evoked potentials have emerged as a particularly informative approach. These event-related potentials, typically elicited by sudden whole-body perturbations of upright stability, are recorded from mobile electroencephalography and provide insight into cortical processing of multisensory information during postural challenge (Varghese et al., 2017). PEPs are most dominantly observed in fronto-central areas of the brain and consist of several distinct components (P1, N1, P2), each sensitive to environmental and psychological factors such as stimulus magnitude, predictability and salience (Marlin et al., 2014; Varghese et al., 2017, 2019; Purohit and Bhatt, 2022). Notably, PEPs are especially characterized by a prominent N1 component that is considerably larger than the N1 evoked by unimodal sensory stimuli, reflecting the unique convergence of visual, vestibular and somatosensory inputs during whole-body perturbations (Varghese et al., 2017). A range of experimental paradigms have been developed to evoke whole-body PEPs, including support-surface translations or rotations, treadmill accelerations, sternal-nudge perturbations, weight-release and lean-and-release paradigms or seated perturbations (Ackermann et al., 1986; Duckrow et al., 1999; Staines et al., 2001; Quant et al., 2004a; Adkin et al., 2006, 2008; Mochizuki et al., 2010; Sibley et al., 2010; Marlin et al., 2014; Ditz et al., 2020). Collectively, PEPs were shown to be valuable for probing the detection of changes in the ongoing postural state and processing of perturbation characteristics by the cerebral cortex, but were also controversially discussed to reflect postural error detection, proprioceptive mismatch or motor reafference (Purohit and Bhatt, 2022; Jalilpour and Müller-Putz, 2023). However, despite a substantial body of research (Varghese et al., 2017), the precise neurophysiological mechanisms underlying these distinct cortical responses and their functional role in postural control are still to be elucidated. While such models have advanced the understanding of the temporal coupling between whole-body sensory input and motor output, they tend to conceptualize PEPs as downstream elements of reactive postural control rather than as part of a broader cortical mechanism. Consequently, the interpretation of PEPs has remained confined to task-specific feedback loops, without fully addressing whether these distinct signatures may simply represent a generalized neural mechanism for the fast registration of sudden changes in sensory state and preparation for potential motor reactions.

One indication for such a mechanism is that reactive postural corrections are typically initiated at latencies that are shorter than those of the fastest stimulus-triggered voluntary movements, yet they occur later than the short-latency spinal stretch reflex (Abbruzzese et al., 1985; Matthews, 1991). This intermediate timing implies that the underlying control is not confined to segmental spinal circuitry but likely incorporates processing in supraspinal pathways, leaving greater scope for context-dependent modulations (Jacobs and Horak, 2007). Moreover, although the PEP is considered a biomarker of whole-body balance function (Mirdamadi et al., 2024), this characteristic potential could also be evoked through isolated single-joint perturbations. (Quant et al. 2004a), for instance, used an inverted pendulum at the ankle joint/shank to elicit PEPs in a seated position, while other studies successfully evoked similar cortical responses using a manipulandum at the wrist (Spieser et al., 2010; Campfens et al., 2015; Govender et al., 2024). Therefore, (Quant et al. 2004a) already suspected at the time that the perturbation-evoked N1 might be a cortical representation of perturbation-related afferent inflow, rather than neural activity specifically associated with initiating or scaling an active balance-corrective response. As most of these isolated limb paradigms minimized head and neck motion, it could further be inferred that vestibular and cervicoceptive contributions were negligible and the PEP N1 is thus rather likely attributed to lower limb proprioceptive and/or cutaneous afference triggered by sudden external disturbance. Consequently, the neural PEP signature would reflect, at least in part, a more general response of the brain to abrupt sensory surprise, rather than representing a signal specific to the ensuing whole-body postural correction alone.

The proposed sensory change-detection interpretation is further reinforced by a striking similarity between the PEP and the VP with respect to their morphological and topographical properties. Analogous to the PEP known from balance studies, the VP is a stereotyped, biphasic, large-amplitude event-related potential maximal at the midline fronto-central scalp (Cz) that can be evoked by sudden, salient stimuli across sensory modalities, including nociceptive, tactile, auditory and visual events (Buchwald et al., 1981; Kraus et al., 1995; Somervail et al., 2022). It is typically expressed as a prominent negative–positive complex occurring roughly ~100–300 ms after stimulus onset and is thought to reflect supramodal processes such as salience detection rather than modality-specific sensory encoding alone. Because the VP is strongly modulated by several stimulus factors, it is widely used as an index of the brain's response to abrupt changes in the sensory environment (Davis, 1939; Walter, 1964; Hillyard and Picton, 1978; Somervail et al., 2022, 2025). Interestingly, the VP and the PEP share similar characteristics of the evoking event (Table 1). The most fundamental and predominantly shared feature is that both exogeneous potentials are provoked by abrupt sensory stimulation contingent on sufficient novelty (Varghese et al., 2017; Somervail et al., 2025). As such, these neural representations are modulated by the saliency, unpredictability, intensity and behavioral relevance of the sensory event (Beydoun et al., 1993; Staines et al., 2001; Adkin et al., 2008; Mouraux and Iannetti, 2009; Sibley et al., 2010; Spieser et al., 2010; Ronga et al., 2013; Moayedi et al., 2016). While the PEP is elicited by sudden external mechanical displacement of single or multiple joints (Dimitrov and Gavrilenko, 1996; Duckrow et al., 1999; Staines et al., 2001; Quant et al., 2004a; Adkin et al., 2008; Mochizuki et al., 2009; Varghese et al., 2017; Allexandre et al., 2019; Ditz et al., 2020; Jalilpour and Müller-Putz, 2023), VPs could be induced by supramodal instantaneous visual, auditory, somatosensory or nociceptive cues (Somervail et al., 2021, 2025). Notably, the concept of a sudden and isolated sensory stimulus as the trigger of VP generation (Somervail et al., 2025) appears to apply equally well to externally imposed mechanical and sensory perturbations, highlighting a potential conceptual convergence between PEP- and VP-evoking stimuli. Moreover, upon repeated stimulation and consequently reduced novelty, both potentials habituate rapidly, showing a progressive amplitude reduction in their negative and positive components (Fruhstorfer, 1971; Iannetti et al., 2008; Mouraux and Iannetti, 2009; Mierau et al., 2015; Ethridge et al., 2016; Novembre et al., 2018; Payne et al., 2019; Somervail et al., 2022; Mirdamadi et al., 2024).

**Table 1:** Shared characteristics of the perturbation-evoked potential (PEP) and vertex potential (VP).

Domain and feature	PEP	VP
Spatiotemporal profile		
Localization (vertex)	Mochizuki et al., 2009 **(FCz)** Marlin et al., 2014 **(BA 6)** Varghese et al., 2017 **(FCz/Cz)** Varghese et al., 2019 **(FCz)** Purohit and Bhatt, 2022 **(FCz/Cz)** Mierau et al., 2015 **(Cz/BA6)**	Walter, 1964 **(vertex)** Buchwald et al., 1981 **(vertex)** Kraus et al., 1995 **(~Fz)** Mouraux and Iannetti, 2009 **(Cz)** Somervail et al., 2021 **(Cz)**
Morphology	Varghese et al., 2017 **(P1-N1-P2 complex)** Purohit and Bhatt, 2022 **(P1-N1-P2 complex)**	Hillyard and Picton, 1978 **(N1-P2 complex)** Mouraux and Iannetti, 2009 **(N1-P2 complex)** Somervail et al., 2025 **(P1-N1-P2 complex)**
Stimulus features		
Salience, novelty, surprise	Adkin et al., 2008 **(↑predictability—↓N1)** Sibley et al., 2010 **(↑postural threat—↑N1)** Mirdamadi et al., 2024 (**↑predictability—↓N1**)	Hillyard and Picton, 1978 **(↑novelty—↑N1)** Spieser et al., 2010 **(↑anticipation—↓N1)** Ronga et al., 2013 **(↑salience—↑N1)** Moayedi et al., 2016 **(↑salience—↑N1)** Kilintari et al., 2018 **(↑salience—↑N1)** Somervail et al., 2022 **(↑surprise—↑N1)**
Supramodality	Ackermann et al., 1986 **(platform tilt)** Dimitrov and Gavrilenko, 1996 **(platform translation)** Duckrow et al., 1999 **(platform rotation)** Staines et al., 2001 **(seated translation)** Quant et al., 2004a,b **(seated pendulum tilt)** Adkin et al., 2006, 2008 **(trunk push)** Mochizuki et al., 2010 **(lean and release)** Barnett-Cowan et al., 2010 **(seated translation)** Ditz et al., 2020 **(seated lateral tilt)** Jalilpour and Müller-Putz, 2023 **(seated lateral tilt)**	Mouraux and Iannetti, 2009 **(nociceptive/non-nociceptive – somatosensory/auditory/visual)** Somervail et al., 2021 and 2022 **(auditory/somatosensory)**
Intensity	Staines et al., 2001 **(↑acceleration—↑N1)** Mochizuki et al., 2010 **(↑displacement—↑N1)** Payne et al., 2019 **(↑acceleration—↑N1)**	Beydoun et al., 1993 **(↑pain—↑N1)** Iannetti et al., 2008 **(↑pain—↑N1)** Somervail et al., 2021 **(↑auditory—↑N1)**
Habituation	Mierau et al., 2015 **(↓N1, 10 trials)** Payne et al., 2019 **(↓N1, 64 trials)** Mirdamadi et al., 2024 **(↓N1, 2 trials)**	Fruhstorfer, 1971 **(↓N1, 7 trials)** Iannetti et al., 2008 **(↓N1, 3 trials)** Ethridge et al., 2016 **(↓N1, 4 trials)**
Clinical usefulness		
Sensitivity to factors	Duckrow et al., 1999 **(↓N1 in elderly)** Ozdemir et al., 2018 **(↓N1 in elderly)** Allexandre et al., 2019 **(↓N1 in TBI patients)** Payne et al., 2022 **(↑N1 with** **↓balance confidence)**	Ethridge et al., 2016 **(↑N1 in FXS)** Miyakoshi et al., 2025 **(↑N1 in FXS)**

FCz/Cz/Fz, electrode positions; BA, Brodmann area; P1/N1/P2, event-related potential components; TBI, traumatic brain injury patients; FXS, fragile X syndrome patients; **↓** lower or decreased amplitude; **↑** higher or increased amplitude. Bold fonts indicate relevant study outcomes/features that support shared characteristics of the two event-related potentials.

Taken together, the strong phenomenological overlap between PEPs and the VP including latency range, sensitivity to stimulus salience and multimodal sensory trigger events suggests that both responses may engage shared supramodal processes involved in the rapid detection of unexpected sensory change (Benusiglio and Asari, 2024). In this context, a theoretical framework dating back to the 1940s has recently gained renewed attention, positing that sensory thalamo-cortical transmission may comprise two parallel projection systems: a highly specific, topographically organized sensorimotor relays pathway and a more diffuse, widely projecting pathway that modulates the state of cortical networks (Somervail et al., 2025). On the one hand, through the high-precision lemniscal pathway, signals that have been processed in the midbrain are conveyed to a modality-specific thalamic relay nucleus and then forwarded to the corresponding primary sensory cortices, where fine-grained stimulus features are processed. While this lemniscal pathway originally refers to the anatomy of auditory and somatosensory modalities, (Somervail et al. 2025) applied this concept to vision, thereby extending the dual-thalamic concept more generally. Encompassing structures such as the medial/lateral geniculate nucleus and the ventroposterior nuclei, the lemniscal system is characterized by modality-dependent, short-latency responses with high fidelity and sharp tuning. Its responses are comparatively resistant to habituation, relatively insensitive to fluctuations in arousal and typically show little change in amplitude as stimulus rate increases (Somervail et al., 2025). On the other hand, the so-called extralemniscal pathway is organized differently and is thought to subserve non-specific, integrative and multisensory functions. In contrast to the sensory inflow targeting the ventral thalamic relay nuclei, the extralemniscal system projects predominantly to intralaminar, medial and posterior thalamic nuclei, including the centromedian–parafascicular complex and nuclei associated with the geniculate complex. These nuclei respond to stimulation from various peripheral body regions and are considered part of a distributed thalamic network mediating integrative and modulatory aspects of somatosensory processing (Albe-Fessard and Besson, 1973). Despite projecting in a narrowly targeted manner, outputs from this system are distributed widely across extensive cortical territories, likely extending beyond the boundaries of a single sensory area. Functionally, the extralemniscal pathway demonstrates enhanced sensitivity to salient stimulation and is specialized for detecting novel or unexpected events, with responses tending to be supramodal, lower in fidelity, more broadly tuned, longer in latency and with greater trial-to-trial variability allowing to habituate rapidly with repeated stimulation (Miyakoshi et al., 2025; Somervail et al., 2025). Within this framework, the characteristics of PEPs show notable convergence with response properties commonly attributed to extralemniscal thalamo-cortical transmission. Therfore, we hypothesize that PEPs may reflect the consequence of the activation of a supramodal sensory pathway that signals salience, potential threat or sensory urgency during postural destabilization and might run in parallel to the general postural control functions.

**Figure 1:**
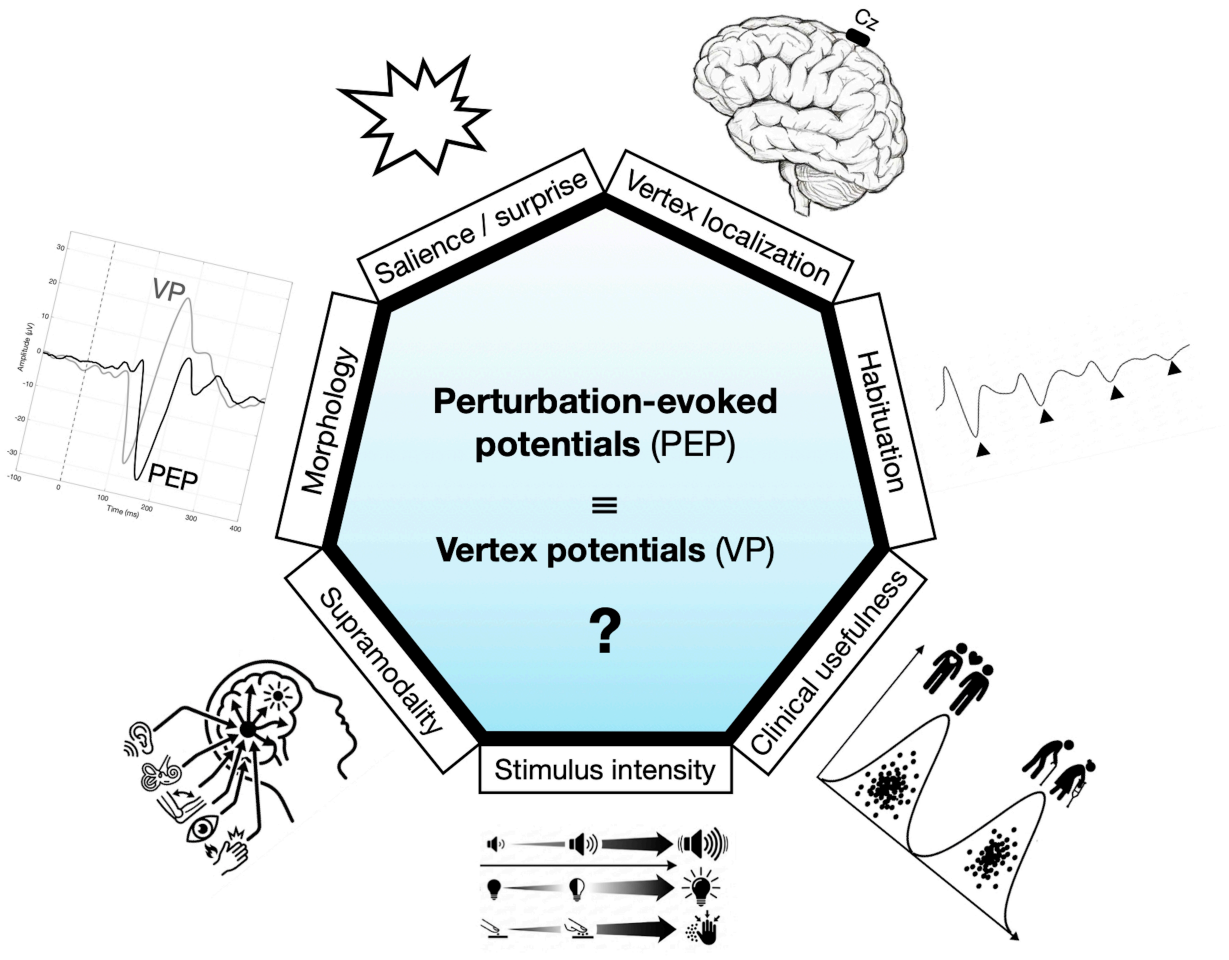
Summary of evidence-based shared characteristics of the perturbation-evoked potential (PEP) and vertex potential (VP), supporting a conceptual overlap between these two neural markers elicited as transient responses to sudden sensory stimulation. The event-related potential waveforms were redrawn and modeled based on previously reported morphologies by (Mirdamadi et al. 2024) for the PEP and (Somervail et al. 2021) for the VP, both sources being licensed under the Creative Commons Attribution 4.0 International License. Changes were made to waveform visualization, labeling, layout and graphical presentation. All other pictograms were generated by Google Gemini (March 2026) based on author-provided specifications and verified by the authors.

Importantly, the present framework (Figure 1) should be regarded as a hypothesis-driven conceptual proposal rather than a validated mechanistic model. Phenomenological similarity between PEPs and vertex potentials alone is insufficient to establish shared neural generators or exclusive pathway involvement. Instead, the proposed interpretation generates testable predictions that motivate further evaluation in future experimental work. To examine this, further studies should directly compare their temporal dynamics, scalp topographies, as well as source characteristics across sensory and postural perturbation modalities. Convergent latencies, vertex-centered distributions and overlapping cortical generators, together with similar habituation and cross-modal refractoriness effects, would ultimately support the interpretation of a common supramodal salience-processing mechanism. Conversely, systematic dissociations in these features would argue for partially distinct functional contributions.

Considering PEPs not only as markers of postural perturbation per se, but also as indicators of sensory transmission and cortical arousal, will open new scientific avenues that may help refine current perspectives in fall prevention, rehabilitation and sports training in the future:

i. **Focus on sensory function:** PEPs might be evaluated for functions beyond just mere read-outs of *how disturbed* postural equilibrium is, extending to dynamic measures of the integrity and efficiency of sensory (ventral thalamic/lemniscal) and alertness/arousal (extralemniscal) transmission pathways (Ozdemir et al., 2018). This information would help to tailor rehabilitation strategies for improving sensory gating, transmission speed or cortical responsiveness, in addition to targeting biomechanical aspects of postural stability.
ii. **Biomarker for sensory readiness and responsiveness:** with their robust and reproducible features, PEPs may serve as neurophysiological markers for the capacity to rapidly transmit salient sensory information to the cortex and to elicit an arousal response. Since degraded sensory transmission or blunted cortical arousal could precede actual motor failure or falls, specific PEP properties should be assessed for their predictive capacity to support early identification of respective vulnerability. This could help identify persons at risk who, even if no overt balance problems could be observed through biomechanical measures, show suboptimal sensory or arousal responses (Lehmann et al., 2021).
iii. **Rethinking rehabilitation success:** in line with the previous aspect, PEP characteristics might also be sensitive indicators of sensorimotor adaptations following exercise interventions, thereby shifting emphasis from traditional biomechanical endpoints (e.g., sway measures or step latency) toward neurophysiological markers (Jacobs, 2014; Payne et al., 2022; Vitharana et al., 2024).

In summary, this opinion article proposes a conceptual shift that emphasizes the role of rapid cortical processing of unexpected sensory input in postural control. By highlighting the phenomenological similarity between PEPs and VPs, we aim to stimulate hypothesis-driven research into whether PEPs reflect supramodal sensory and arousal-related mechanisms in addition to postural control processes. Clarifying this relationship has the potential to refine both experimental approaches and translational perspectives, provided that future work establishes specificity, validity and functional relevance.
